# Double stranded RNA-dependent protein kinase promotes the tumorigenic phenotype in HepG2 hepatocellular carcinoma cells by activating STAT3

**DOI:** 10.3892/ol.2014.2560

**Published:** 2014-09-24

**Authors:** XUN WANG, JIA-HONG DONG, WEN-ZHI ZHANG, JIAN-JUN LENG, SHOU-WANG CAI, MING-YI CHEN, XUERUI YANG

**Affiliations:** 1 Department of Hepatobiliary Surgery, The General Hospital of Chinese People’s Liberation Army, Beijing 100853, P.R. China; 2 MOE Key Laboratory of Bioinformatics, Tsinghua-Peking Center for Life Sciences, School of Life Sciences, Tsinghua University, Beijing 100084, P.R. China

**Keywords:** double stranded RNA-dependent protein kinase, signal transducer and activator of transcription 3, hepatocellular carcinoma, HepG2, tumorigenesis

## Abstract

Previously known as a first-response protein upon viral infection and other stress signals, double-stranded RNA-dependent protein kinase (PKR, also termed EIF2AK2) has been found to be differentially expressed in multiple types of tumor, including hepatocellular carcinoma, suggesting that PKR may be involved in tumor initiation and development. However, whether and how PKR promotes or suppresses the development of hepatocellular carcinoma remains controversial. In the present study, PKR expression was investigated using qPCR and western blot analysis, which revealed that PKR expression was upregulated in liver tumor tissues, when compared to that of adjacent normal tissues, which were obtained from four primary liver cancer patients. Furthermore, *in vitro* cellular assays revealed that PKR exerts a key role in maintaining the proliferation and migration of HepG2 human hepatocellular carcinoma cells. Mouse models with xenograft transplantations also confirmed a tumorigenic role of PKR in HepG2 cells. Furthermore, a transcription factor, signal transducer and activator of transcription 3 (STAT3), was revealed to mediate the tumor-promoting function of PKR in HepG2 cells, as shown by *in vitro* cellular proliferation and migration assays. In conclusion, the results suggested a tumorigenic role of PKR in liver cancer and a detailed mechanism involving an oncogenic transcription factor, STAT3, is described. Therefore, PKR may present a potential novel therapeutic target for the treatment of liver cancer.

## Introduction

Double-stranded RNA-dependent protein kinase (PKR, also known as EIF2AK2) was originally identified as a first-response protein, which induces cell defense responses upon viral infection (1). Briefly, double-stranded RNA, usually produced during virus replication by viral RNA polymerases, binds protein PKR, facilitates the homo-dimerization and auto-phosphorylation of PKR at Thr451 and Thr446, and thereby activates PKR (2,3). PKR has also been found to be activated by other stress signals, therefore serving as a signaling hub of the proinflammatory response to stimuli including bacterial lipopolysaccharide, tumor necrosis factor α and interleukin 1 (4). In the canonical PKR signaling pathway, PKR serves as a eukaryotic initiation factor 2α (eIF-2α) kinase, which promotes the phosphorylation of eIF-2α at Ser51 (3). Phosphorylated eIF-2α inhibits the initiation of translation, resulting in the suppression of general protein synthesis and therefore suppression of cell growth and induction of cellular apoptosis, in numerous types of eukaryotic cell (3,5). Therefore, PKR has been previously suggested to be a tumor suppressor due to its potential for inhibiting cell growth and inducing apoptosis (6–8). For example, in liver cancer cells, PKR-activating agents, such as interferon and radicicol, were shown to enhance the apoptotic effect of the transcription factor E2F1, a process proposed to be mediated by transcriptionally upregulated PKR expression (8). However, these effects were marginal, and there remains a lack of direct evidence and detailed mechanisms to show the exact role of PKR in liver cancer development.

Multiple studies have observed increased expression levels and elevated activity of PKR in hepatitis C virus (HCV)-related and -unrelated hepatocellular carcinoma (9–11), as well as in several other cancer cell types, for example, human breast cancer cells (12) and melanoma cells (13,14). Elevated PKR expression levels and activity may be a tumor marker, and may contribute to the proliferation of tumor cells and tumor development (9,12–14). Studies have also suggested that PKR may suppress apoptosis by activating the nuclear factor κB (NF-κB) signaling pathway (15) and the corresponding target gene, B-cell lymphoma 2 (Bcl2), an antiapoptotic protein (16). However, this remains indirect evidence, although this does suggest a potential tumorigenic role of PKR in hepatocellular carcinoma. In addition, although the positive effect of PKR on the antiapoptotic pathway through NF-κB and Bcl2 has been demonstrated, whether PKR exerts any effect on pro-proliferation transcriptional pathways remains unknown.

PKR has been found to activate several transcription factors, including IRF-1, p53 and NF-κB (17,18). PKR has also been shown to directly bind with STAT3 and regulate STAT3 transcriptional activity, although whether PKR activates or suppresses STAT3 remains controversial (19,20). Elevated STAT3 activity, which depends on phosphorylation, has been observed in primary liver cancer (21). Hepatocyte-specific STAT3-deficient mice exhibited markedly greater resistance to hepatocellular carcinoma and the tumor sizes were clearly smaller, suggesting that STAT3 is crucial in promoting hepatocellular carcinoma cell proliferation and/or survival (22).

The present study aimed to provide direct evidence with regard to the function of PKR in liver cancer tumorigenesis via *in vivo* and *in vitro* assays, and to describe the detailed underlying mechanism. Furthermore this study aimed to clarify the oncogenic role of PKR in hepatocellular carcinoma and detail the mechanism.

## Materials and methods

### Patients and tissues

Tumor and adjacent normal tissue samples were obtained from four primary liver cancer patients (two males and two females) at the Department of Hepatobiliary Surgery, The General Hospital of Chinese People’s Liberation Army (Beijing, China) in 2012. The patient’s ages ranged between 50 and 60 years. The four patients were first-time diagnosed with primary liver cancer, without Hepatitis B or C virus infection. The normal and tumor samples were collected during the first surgical procedures performed in 2012, subsequent to diagnosis, and were freshly cryo-preserved in liquid nitrogen. The frozen tissue samples were grinded using a Spex 6770 Freezer/Mill system (SPEX SamplePrep LLC, Metuchen, NJ, USA). This study was approved by the ethics committee of The General Hospital of Chinese People’s Liberation Army and written informed consent was obtained from all patients.

### Cell culture and reagents

HepG2 human hepatocellular carcinoma cells (American Type Culture Collection, Manassas, VA, USA) were cultured in Dulbecco’s modified Eagle’s Medium (cellgro^®^; Mediatech, Inc., Manassas, VA, USA) with 10% fetal bovine serum (FBS; Thermo Fisher Scientific, Rockford, IL, USA). Freshly trypsinized HepG2 cells were suspended at 1×10^5^ cells/ml in standard HepG2 culture medium and seeded at a density of 2×10^4^ cells per well in standard 24-well tissue culture plates. Subsequent to seeding, the cells were incubated at 37°C in a 90% air/10% CO_2_ atmosphere, and 500 μl fresh medium was supplied every other day to the cultures following removal of the supernatant.

### Small interfering RNA (siRNA)-mediated RNA interference and reverse transfection

Silencer^®^ Select Validated siRNA targeting human PKR and STAT3 was purchased from Ambion (Austin, TX, USA). The synthesized oligonucleotides were as follows: PKR siRNA, sense, 5′-GGUGAAGGUAGAUCAAAGATT-3′ and anti-sense, 5′-UCUUUGAUCUACCUUCACCTT-3′; STAT3 siRNA, sense, 5′-GCACAATCTACGAAGAATCAATT-3′ and anti-sense, 5′-TTGATTCTTCGTAGATTGTTT-3′. As described previously (16), transfection of siRNA was performed with Lipofectamine RNAiMAX transfection reagent (Invitrogen Life Technologies, Carlsbad, CA, USA). Scrambled non-targeting siRNA served as a negative control. Titration of the siRNA and the transfection reagent was performed (not shown), and the lowest viable siRNA and transfection reagent quantities were subsequently applied in the loss-of-function (LOF) experiments.

### Lentivirus-mediated RNA interference

Short hairpin RNA (shRNA) sequences were obtained from Public TRC Portal (Broad Institute, Cambridge, MA, USA) and lentiviruses expressing the shRNA sequences were synthesized by Shanghai GenePharma Co., Ltd., (Shanghai, China). Polybrene and puromyocin were purchased from Sigma-Aldrich (St. Louis, MO, USA). Transfection of the HepG2 cells with lentiviral particles (Shanghai GenePharma Co., Ltd.)was conducted as described previously (23). Titration of the lentiviruses was performed (not shown), and the lowest functional quantities of the virus (MOI=5) and polybrene were subsequently applied in the LOF experiments. The shRNA target sequences for PKR were as follows: shPKR-1, 5′-GCTGAACTTCTTCATGTATGT-3′ and shPKR-2, 5′-GAGGCGAGAAACTAGACAAAG-3′. The shRNA target sequence for STAT3 was 5′-GCACAATCTACGAAGAATCAA-3′.

### Overexpression of PKR and forward transfection

A pCMV6-XL5-hPKR plasmid and an empty vector, pCMV6-XL5, were purchased from Origene (Rockville, MD, USA). As described previously (16), forward transfection of the plasmid was performed with the Lipofectamine 2000 (Invitrogen Life Technologies) transfection reagent, following the manufacturer’s instructions. The cells attached to the culturing surface were washed with phosphate-buffered saline (PBS) and the medium was replaced with 100 μl Opti-MEM^®^ (Invitrogen Life Technologies) with 2% fetal bovine serum. Subsequently, 400 ng plasmid per well in a 24-well plate were mixed with 1 μl/well Lipofectamine 2000 in Opti-MEM and, 20 min later, the mixture was added to the cells. After 6 h of transfection, the cells were cultured in regular medium for 24 h and subsequently harvested.

### Western blot analysis

The HepG2 cells or tissue samples were lysed as described previously (16) with RIPA buffer (Cell Signaling Technology, Inc., Beverly, MA, USA). Plated cells or cryo-grinded tissue samples were incubated with RIPA lysis buffer (Cell Signaling Technology, Inc.) for 10 min, then collected and sonicated briefly. Next the samples were centrifuged at 14,000 × g for 10 min in a cold microfuge (5424R; Eppendorf, Hamburg, Germany). Total protein levels were quantified using a bicinchoninic assay kit from Pierce Biotechnology, Inc. (Rockford, IL, USA). Subsequently, 20–40 μg total protein was resolved using SDS-PAGE gels (Bio-Rad, Hercules, CA, USA), transferred to nitrocellulose membranes, and then probed with primary antibodies (1:1,000) overnight and secondary antibody (1:5,000) for 1 h. Biotinylated protein ladders (Cell Signaling Technology, Inc.) were loaded onto one well of each SDS-PAGE gel and anti-biotin antibody was employed to detect these protein ladders on western blots. An enhanced chemiluminescence kit (Pierce Biotechnology, Inc.) was used for the antibody detection, and images were captured using the Molecular Imager ChemiDoc XRS system (Bio-Rad). Monoclonal rabbit anti-human STAT3 (Tyr705), monoclonal mouse anti-human STAT3 and polyclonal rabbit anti-human STAT3 (Ser727) antibodies were purchased from Abcam (Cambridge, MA, USA), and monoclonal mouse anti-β-actin, polyclonal rabbit anti-PKR and polyclonal rabbit anti-PKR (Thr451) antibodies were obtained from Sigma-Aldrich. Horseradish peroxidase-conjugated secondary polyclonal goat anti-rabbit and anti-mouse antibodies were purchased from Pierce Biotechnology, Inc.

### Reverse transcription quantitative polymerase chain reaction (RT-qPCR) analysis

Total RNA was extracted from the cells and tissue samples with an RNeasy mini kit (Qiagen, Valencia, CA, USA) and was depleted of contaminating DNA with RNase-free DNase (Qiagen). Equal quantities of total RNA (1 μg) were reverse-transcribed using a High Capacity cDNA Reverse Transcription kit (Applied Biosystems, Carlsbad, CA, USA). The first-strand cDNA served as a template. The primers used for RT-qPCR analyses were as follows: Human PKR, forward, 5′-ACTTTTTCCTGGCTCATCTC-3′ and reverse, 5′-ACATGCCTGTAATCCAGCTA-3′; and human GAPDH, forward, 5′-AACTTTGGTATCGTGGAAGGA-3′ and reverse, 5′-CAGTAGAGGCAGGGATGATGT-3′, and were synthesized by Invitrogen Life Technologies. qPCR was performed as described previously (16). The SYBR® Select Master Mix (Applied Biosystems, Carlsbad, CA, USA) was used in 20 μl PCR reaction systems. Ct values <30 were considered to be reliable in the assay. The human PKR expression levels were normalized to those of GAPDH.

### Cell proliferation assay

Experiments were conducted in an xCELLigence Real-Time Cell Analyzer (RTCA) DP system (Roche, Mannheim, Germany). The cells were seeded in 16-well plates (4,000 cells in 150 μl medium/well; E-plate 16; Roche), according to the manufacturer’s instructions. The cell index, which is proportional to the number of cells attached to the culturing surface, was recorded in real-time every 1–2 h for up to 3–4 days. For each well, the cell index recorded 4 h after seeding served as the baseline to subsequently obtain the cell index fold changes. The time point of 4 h after seeding was therefore used to indicate time point zero in Fig. 2A. The average fold changes in the cell proliferation index were calculated from at least four replicate experiments, and are shown as the mean ± standard error (SE).

### Cell migration assay

Cell migration experiments were conducted in the xCELLigence RTCA DP system (Roche). The cells were suspended in serum-free medium and seeded in the upper chambers of 16-well CIM-Plate 16 plates (40,000 cells in 150 μl medium/well; Roche). Regular medium with 10% FBS was added to the lower chamber of the CIM-Plate 16. The experiment setting and plate design were similar to those of conventional Transwell migration assays. The cell index, which is proportional to the number of cells that migrate through the pores of the upper chamber, was recorded in real-time every 30 min for up to 24 h. The average cell migration index was calculated from at least four replicate experiments, and is presented as mean ± SE.

### In vivo xenograft transplantation assay

HepG2 cells growing exponentially *in vitro* were trypsinized and harvested for tumor implantation. Male 6–8 week old nude CD-1 mice were purchased from Vital River Laboratory Animal Technology Co., Ltd., (Beijing, China). For each condition, five mice were injected subcutaneously in the right flank with 2×10^6^ HepG2 cells in 0.1 ml PBS with 0.5% bovine serum albumin (MP Biomedicals, Santa Ana, CA, USA). When tumors became visible, the tumor volume was monitored every three days using caliper measurements and was calculated by the following formula: Tumor volume (mm^3^) = tumor length (mm) × tumor width (mm)^2^ / 2. At 27 days after injection, the animals were sacrificed using CO_2_, and the xenografted tumors were isolated and weighed.

### Statistical analysis

All experiments were performed at least three times and representative results are shown. Data are presented as the mean ± SD for the indicated number of experiments, unless specified otherwise. One-way analysis of variance with Student’s t-test was used to evaluate statistical significances amongst the different treatment groups. P<0.05 was considered to indicate a statistically significant difference.

## Results

### PKR is upregulated in hepatocellular carcinoma tumor tissue samples

**Figure 1:**
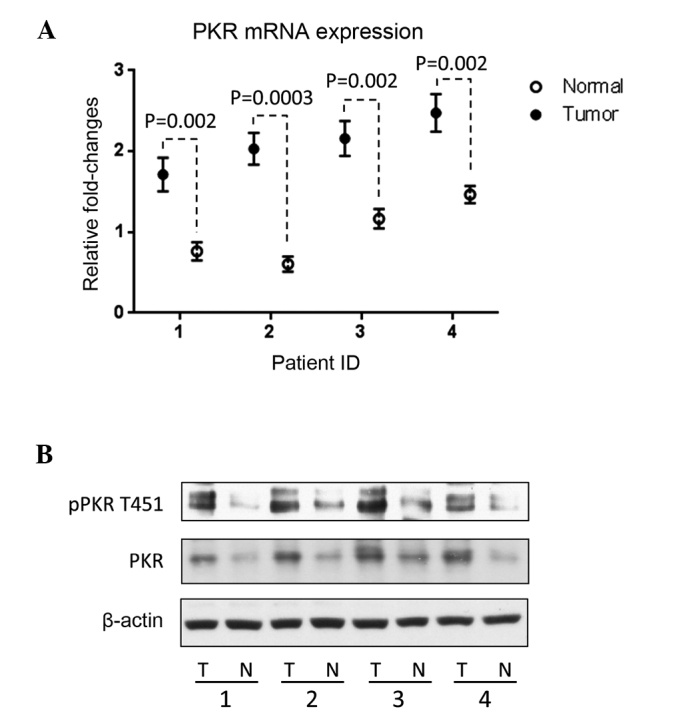
Expression levels of RNA-dependent protein kinase (PKR) in liver tumor tissues and adjacent normal tissues. Liver tumor tissue samples were collected from four hepatocellular carcinoma patients, and the (A) mRNA expression of PKR, as well as (B) protein expression of PKR and phosphorylation of PKR, were measured. (A) Relative fold changes of PKR mRNA expression levels, measured by reverse transcription quantitative polymerase chain reaction, in the liver tumor tissue and normal tissue samples. Error bars signify standard deviations (+/-) of three independent tests of tissue samples taken from the same patient. (B) PKR total protein and phosphorylation at Thr451 levels in tumor (T) and normal (N) tissue samples from patients 1–4 were measured with western blotting. β-actin served as an internal reference.

qPCR and western blotting were performed to measure the PKR mRNA and protein expression levels respectively in tumor tissues, using the adjacent normal tissue as a reference. PKR mRNA (Fig. 1A) and protein expression (Fig. 1B) was upregulated in all four tumor samples, compared with the adjacent normal tissues. PKR protein activity depends on phosphorylation at Thr451 (2,3); therefore, the phosphorylation level of PKR at Thr451 was also measured in all four tumor tissue samples. The results revealed that the PKR protein activity, indicated by the phosphorylation level at Thr451, was also higher in the liver tumor samples than the normal tissues (Fig. 1B). Statistically significant differences in PKR mRNA expression between normal and tumor tissues were identified in all four patients (P=0.002, P=0.0003, P=0.002 and P=0.002, respectively). These results confirmed those of previous reports, which observed elevated PKR expression levels in tumor tissues, including tissues from liver tumors, and which revealed that the total phosphorylation of PKR is also higher in tumor tissues (9–14). As determined by these findings, the potential function of PKR in regulating tumor cell phenotype, for instance, in modifying proliferation and migration, was further analyzed.

### PKR is involved in maintaining liver cancer cell proliferation and migration

**Figure 2:**
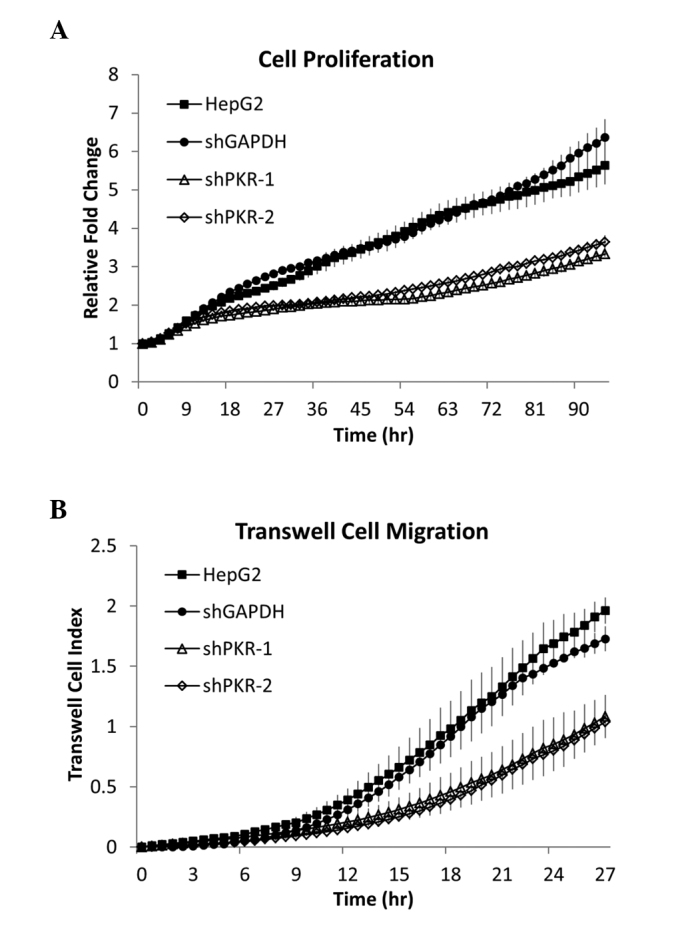
Involvement of RNA-dependent protein kinase (PKR) in maintaining cell proliferation and migration. (A) Cell proliferation and (B) Transwell migration were recorded in real-time in HepG2 human hepatocellular carcinoma cells in which PKR had been knocked down. Error bars indicate standard errors of four replicates.

HepG2 cells served as a model for hepatocellular carcinoma. Silencing PKR gene expression with PKR shRNA markedly reduced the proliferation rate of HepG2 cells (Fig. 2A), suggesting that PKR is involved in promoting HepG2 cell proliferation. In addition, as cell migration is an early requirement for tumor metastasis and the rate of migration indicates the aggressiveness of cancer cells, the effect of PKR on cell migration was examined with *in vitro* Transwell migration assays. Silencing PKR with shRNA markedly suppressed HepG2 cell Transwell migration (Fig. 2B). To further investigate the role of PKR in promoting HepG2 cell proliferation and Transwell migration, PKR expression following gene silencing was rescued. Proliferation and migration were completely restored by rescuing PKR expression in HepG2 cells (Fig. 4B and C). The results indicate that PKR is central in promoting and maintaining HepG2 cell proliferation rates and migration through micrometer pores. Thus, PKR may be involved in maintaining liver cancer cell proliferation and migration, suggesting a potential tumorigenic role for PKR in liver tumor cells.

### PKR is involved in liver cancer tumorigenesis

**Figure 3:**
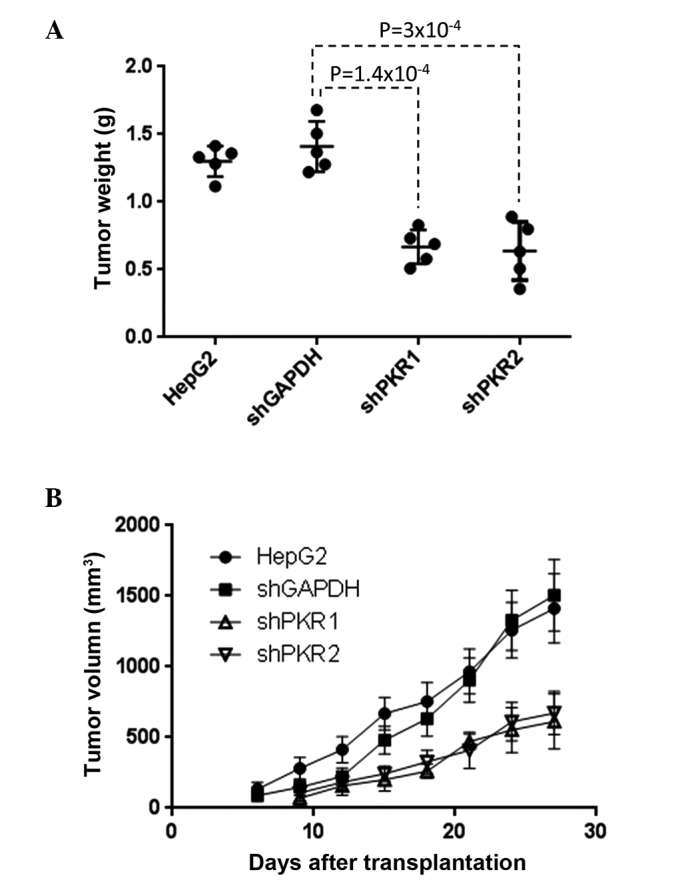
Tumorigenic role of RNA-dependent protein kinase (PKR) in HepG2 human hepatocellular carcinoma cells. (A) Weight and (B) volume of tumors following the transplantation of control HepG2 cells and HepG2 cells in which PKR had been knocked down. (A) Weight of xenografted tumors 27 days after transplantation. The data from five individual mice are shown as scattered dots, along with the average weight and standard errors. (B) Tumor volume was measured every three days. Error bars signify the standard error of five biological replicates.

To examine the role of PKR in tumorigenesis *in vivo*, xenograft transplantation experiments were performed in mice. Vector-based PKR shRNA was used to prepare the HepG2 cell line that stably expresses shPKR and therefore silences PKR long-term. These cells were then subcutaneously injected into nude mice (CD1^−/−^). Compared with the transplantation of regular HepG2 cells and HepG2 cells that stably expressed empty vector, transplantation of the cells that stably expressed shPKR resulted in markedly slower tumor growth and smaller tumor size four weeks after transplantation (Fig. 3). This clearly demonstrates that PKR exerts an important role in promoting tumor development *in vivo*.

Overall, we have shown that PKR, which is upregulated in primary liver tumors, is involved in maintaining HepG2 cell proliferation and migration, and also exert a key role in HepG2 cell tumorigenesis *in vivo*. However, the mechanisms by which PKR regulates cell proliferation and migration, as shown in Fig. 2, remains unclear.

### PKR mediates HepG2 cell proliferation and migration through STAT3

Previous studies have identified multiple downstream targets of PKR, including eIF-2a, NF-κB and c-Jun N-terminal kinase (3,15–17). Silencing PKR has been shown to reduce Bcl-2 expression levels through NF-κB. This was suggested to be the mechanism of PKR-regulated cellular apoptosis. Indeed, the NF-κB signaling pathway has been shown to be a key regulator of human hepatocellular carcinoma development (24,25), through various mechanisms. In addition to NF-κB, other transcription factors, such as STAT3, have also been suggested to promote the development of liver cancer (26). High STAT3 activity levels, which depend on STAT3 phosphorylation, have been observed in primary liver cancer (21). In addition, hepatocyte-specific STAT3-deficient mice exhibited markedly greater resistance to hepatocellular carcinoma and the tumor sizes were evidently smaller, suggesting that STAT3 is crucial in promoting hepatocellular carcinoma cell proliferation and/or survival (22). Notably, PKR has been demonstrated to directly bind with STAT3 and regulate STAT3 transcriptional activity, although whether PKR activates or suppresses STAT3 remains controversial (19,27).

**Figure 4:**
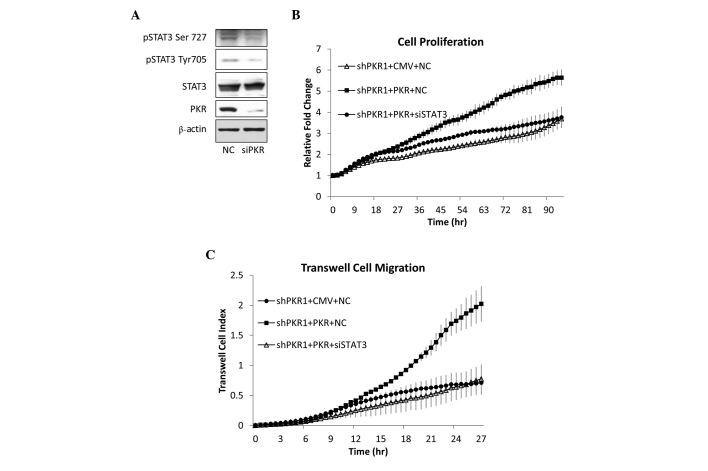
Involvement of STAT3 in mediating the effects of RNA-dependent protein kinase (PKR) in cell proliferation and migration. (A) Total protein expression levels of STAT3 and PKR, as well as the STAT3 phosphorylation levels at Ser727 and Tyr705, were measured with western blotting. β-actin served as an internal reference. (B) Cell proliferation and (C) Transwell migration were recorded in real-time in HepG2 human hepatocellular carcinoma cells with PKR knockdown, PKR rescue and PKR rescue + STAT3-silencing. Error bars indicate standard errors of four replicates.

In the present study, the effect of PKR on phosphorylation of STAT3 was analyzed. Silencing PKR gene expression in HepG2 cells with siRNA reduced STAT3 phosphorylation at Tyr705 and Ser727 (Fig. 4A). Therefore, in HepG2 hepatocellular carcinoma cells, PKR positively regulates STAT3 phosphorylation, a process hypothesized to determine the activity of STAT3. Whether STAT3 is involved in mediating the positive effect of PKR in promoting tumor cell growth was then investigated. As shown in Fig. 2, shRNA lentivirus-mediated PKR gene silencing markedly reduced HepG2 cell proliferation and migration. Subsequent rescue of PKR expression following PKR knock-down restored cell proliferation and migration (Fig. 4B and C). Furthermore, in PKR-restored HepG2 cells, STAT3 expression was silenced with STAT3 siRNA. This completely reversed the effects of rescuing PKR expression on cell growth and migration rates (Fig. 4B and C). These results demonstrate that PKR is essential in maintaining the high growth and migration rates of HepG2 cells, and that this effect depends on a well-known oncogenic transcription factor, STAT3.

## Discussion

Previously, PKR has been recognized as a first-response protein upon viral infection, due to its activation by double-stranded RNA, which initiates innate immune responses by arresting general protein synthesis and inducing apoptosis during viral infection (28). Studies in other systems have revealed further important roles of PKR in mediating multiple signaling pathways, such as NF-κB, mitogen-activate protein kinases (MAPKs) and protein phosphatase 2A (PP2A) (29–32). Therefore, PKR was suggested to exert a key role in other diseases systems, including those of cancer. Due to its function in phosphorylating eIF-2α and thereby inhibiting general protein synthesis (3,5), PKR has been suggested to act as a tumor suppressor by suppressing cell growth and inducing apoptosis (6,7). However, studies have shown that PKR may exert an antiapoptotic role in tumor cells (15,16). Notably, the protein expression levels and activity of PKR have been found to be upregulated in tumor cells; for example, in human breast cancer (12), melanoma (13) and hepatocellular carcinoma cells (9–11). However, few studies investigating the function of PKR in tumor cell proliferation and migration have been published. PKR may suppress cell proliferation (6,33), but the exact effect and the underlying mechanism remain unknown. More recent results in HCV-related HCC revealed that PKR promotes tumor cell proliferation through c-Fos and c-Jun signaling (34). In the present study, the function of PKR in promoting the cell proliferation, migration and, furthermore, tumorigenesis of hepatocellular carcinoma cells was demonstrated. The results also revealed that PKR activates STAT3, a transcription factor associated with primary liver tumors, which is suggested to promote tumor cell proliferation (21).

As a Ser/Thr protein kinase, PKR is able to mediate multiple important signaling pathways (4) in addition to eIF-2α, by interacting with proteins, such as NF-κB, MAPKs and PP2A (29–32). Two independent studies have reported opposite effects of PKR on STAT3 activity (19,20). In mouse embryonic fibroblasts, PKR was shown to dephosphorylate STAT3 at Tyr705 by activating T-cell protein-tyrosine phosphatase (20), while in another study, STAT3 phosphorylation at Tyr705 and Ser727 were observed to be dependent on PKR and the corresponding downstream target ERK (19). This controversy has not been fully resolved, although the effect on phosphorylation may be associated with the basal levels of PKR and STAT3 (20). In the present study, relatively middle-to-high PKR and STAT3 activity levels were observed in HepG2 cells (Fig. 4A), and silencing PKR resulted in reduction of STAT3 activity.

Previous results have also suggested an antiapoptotic role of PKR in HepG2 cells (16). In the present study, by focusing on a small area in which none of the cells undergo apoptosis during the observation time, the non-apoptotic cells exhibited slower proliferation. In addition, the overexpression of PKR by transfecting the pCMV-PKR plasmid into HepG2 cells increased the rate of proliferation, during which no apoptosis was observed in either control or PKR-overexpressing cells. PKR as a protein kinase, activates several transcription factors, including IRF-1, p53 and NF-κB (17,18). The effect of PKR on apoptosis of hepatocellular carcinoma cells depends on the transcription factor NF-κB (16). Notably, multiple transcriptional events have been identified in liver cancer, including NF-κB and STAT3. Previous studies observed that STAT3 and NF-κB activation are mutually exclusive in liver cancer tissues, and the molecules are engaged in positive and negative crosstalk (22,26). Considering the results of previous studies, together with those of the current study, PKR may positively regulate the two transcription factors in the same cell context. Since the factors have different and potentially complementary effects on tumor cell activities, including apoptosis, proliferation and migration, further investigation into whether and how PKR is involved in the crosstalk between NF-κB and STAT3 may be required.
